# The Book World of Medicine and Science

**Published:** 1894-11-17

**Authors:** 


### The Book World of Medicine and Science.

Medical History from the Earliest Times. A popular history of the healing art. By Edward Theodore Withington, M.A., M.B., Oxon. With two illustrations. (London: The Scientific Press, Limited, 428, Strand, W.C. 1894.)

Mr. Withington has condensed into this moderate-sized volume a truly wonderful amount of information concerning the doctors of past ages, their methods and mistakes, and their solid and permanent contributions to the science of disease. The number of authorities whom the author has consulted in forming his compilation are too numerous to cite here; but it may be said that the book gives an interesting and convenient account of all that is most notable in Hindu, Egyptian, classic, aud mediæval medicine. The only fault that can be found, is that the shortness of the chapters gives a most undeserved aspect of desultoriness to the work. This, however, though it tends to conceal the amount of deep and careful research necessary to produce these brief chapters, will make the work more popular among readers of all classes who cannot fail to gain from it an amount of information that will surprise themselves concerning men whose services and sacrifices in the cause of medicine we do ill to forget.

Clinical Medicine. By Judson S. Bury, M.D., F.R.C.P. (C. Griffin and Co.)

The study of disease by the bed-side has been rightly insisted upon as the only means of making a good and trustworthy physician. He who has gained his knowledge mainly by the expenditure of the midnight oil may be crammed full of facts and theories (most useful in their place), but when in the sick-room must give way to the man whose powers of observation have been trained in noting the various aspects of disease. The author of this work has laid the student who aims at attaining this proficiency under heavy obligations. There must have been a vast amount of time and thought given to its production. While the subject is approached in a scientific manner, and the information it supplies is quite up to date, yet it is at the same time thoroughly practical, and its chief aim is to enable the student to record facts, and to teach him how to interpret their true significance. The examination of all the systems of the body is carefully and systematically gone into. If we might mention any section in particular, it would be that devoted to the nervous system, which is very fully dealt with. It is not to the beginner alone that we can cordially recommend this book, but also to his elder brother busily engaged in the practice of his profession. To the latter its value will be much enhanced by the carefully compiled index with which it is furnished. Illustrations have been supplied with a lavish hand; taken as a whole, they are excellent and most useful. Indeed, we may say that the illustrations form a special feature of the work. If we were disposed to be hypercritical we might take exception to Fig. 215, which is hardly worthy of its place in such a collection. A fairly complete chapter on cutaneous eruptions is from the pen of Dr. Wild, while that on the examination of the larynx is supplied by Dr. Harris. We are pleased to see that common sense views on the dangers of specialism are strongly and plainly enunciated. The student is warned in forcible language against the danger of concentrating his attention upon any particular organ to the neglect of the general condition of the patient. Through carelessness or inattention to this obvious precaution much damage may be done to the reputation of the doctor, and real injury may accrue to the patient.

